# The Sale of Medicated Wines

**Published:** 1916-08-05

**Authors:** L. Campbell-Johnston

**Affiliations:** Woodcote Grove House, Coulsdon, Surrey


### The Sale of Medicated Wines.

*To the Editor of* The Hospital.

Sir,—I note your remarks on this subject in your last issue, but would point out that the Order in Council coming into force on August 7 provides that the label shall state clearly the amount of *proof spirit* contained. "Proof spirit" is a trade term equivalent to 60 per cent. of a 90 per cent. solution of absolute alcohol, and must be entirely misleading to medical men. These wines are sold as medicated preparations, and whatever their merits or demerits may be, the medical standard of absolute alcohol should have been adopted. Apparently this is another specimen of a lawyer's handling of scientific matters which has already made our technical legislation so full of anomalies.—Faithfully yours,

L. Campbell-Johnston.

Woodcote Grove House, Coulsdon, Surrey,
July 26, 1916.

